# Lectures in Reply to the Croonian Lectures for 1854, of Charles West, of London, on the Pathological Importance of Ulceration of the Os Uteri

**Published:** 1855-06

**Authors:** 


*Lectures in Reply to the Croonian Lectures for* 1854, *of Charles West, of London, on the Pathological Importance of Ulceration of the Os Uteri.* By HENRY MILLER, M. D., Professor of Obstetric Medicine in the University of Louisville, etc., etc.

These lectures, originally published in the Western Journal of Medicine, are, as the title indicates, a reply to those of Dr. West, noticed by us last month. Dr. Miller says, and that, perhaps, fairly, that Dr. West has taken refuge behind a subterfuge in assigning the name of ulceration to all abrasions or other trivial alterations of structure at the mouth or neck of the womb. It is evident, on a second glance at the Croonian Lectures, that this is measurably the case. For instance, possibly led away by an unconscious ultraism on this subject, Dr. West speaks of discharges from the body of the uterus as *uterine*, and virtually denies that term to discharges from the cervix.

Dr. West, however, writes like an honest man, and we are unwilling to believe that he has intentionally misrepresented any part of his subject. It is nevertheless certain that this operation has been the occasion of a good deal of bitter feeling in London, and we have been told that Dr. West has been a party in the quarrel. If this be true, it would be very difficult for him to shun all prejudice, however straight-forward might be his intentions.

To leave the issue between men, and come back to Prof. Miller's lectures. Dr. Miller writes with an energy and warm controversial spirit, such as, while it in no degree impairs his argument, gives evidence that he has gone into the subject *con amore.* His lectures will be read with interest and profit by either side. He defends the doctrines of Bennett and Simpson, asserts the importance and frequency of ulceration in this locality, and speaks strongly in favor of the treatment by caustic. He denies, and that with great reason, so far as our information extends, the *curative* influences of rest and hygienic measures in this form of disease, considering them only palliatives. In this, all must concur who have seen the hopeless, bed ridden condition, to which many females are reduced by too long confinement to the horizontal posture in the hope of thereby curing displacements of the womb which are purely dependent on local inflammations and the relaxed conditions produced by them; needing tonics, society, exercise, and vigorous local treatment, rather than enfeebling restraints and mechanical contrivances.

